# Reoperation on an Implant-Supported Restoration in the Maxillary Anterior Region to Correct a Complex Aesthetic Deficit

**DOI:** 10.1155/2022/2956643

**Published:** 2022-08-12

**Authors:** M. M. Figliuzzi, L. Parentela, A. Forte, D. Aiello

**Affiliations:** Health Sciences Department “Magna Graecia”, University of Catanzaro, Catanzaro, Italy

## Abstract

**Introduction:**

In an era in which patients are becoming more and more demanding and in which there are many ways to satisfy their needs, modern implantology must consider the correct management of soft tissues during treatment planning, aiming for both functional and aesthetic rehabilitation while creating a prosthetic construction that is in harmony not only with the natural dentition of the patient but also with their face. The patient who came to our notice had a rehabilitative prosthetic implant on the left central incisor area, which did not satisfy any functional or aesthetic parameter. Furthermore, he presented an altered passive eruption in the contralateral hemiarch.

**Materials and Methods:**

The prosthetic crown was removed, the tissues were studied, and the team decided to proceed with customizing a provisional restoration that would cause the soft tissues to descend. A surgical periodontal procedure was then performed to solve the altered passive eruption condition that was also compromising the aesthetics. In conclusion, a permanent prosthetic crown was fixed into place. *Discussion*. Using a periodontal approach that was both surgical and prosthetic, the patient was rehabilitated correctly regaining both functions and aesthetics. It is of fundamental importance that each step in the procedure is carefully programmed; otherwise, the risk of making mistakes increases and solving the problems becomes less simple or less immediate. In order to do this, one must bear in mind that certain clinical cases can apparently concern just one tooth, yet the mouth must be considered as a whole, both functionally and aesthetically. To perform an optimal implantology, the clinician should be an expert in periodontology so that they can plan and, should it be necessary, perform all the therapeutical options (surgical and nonsurgical) that can lead to the best possible result.

**Conclusions:**

The resolution of this complex clinical case has been documented in order to share useful advice for the resolution of analogous cases. We strongly advise that each proposed procedure be planned meticulously and that the periodontological aspect of the case never be separated from the prosthetic or the implantological aspects since the integration of the periodontal tissues is of vital importance for both the functional and the aesthetic results.

## 1. Introduction

Obtaining a functional as well as an aesthetic result is not always immediate, and it can become challenging for dentists. Rehabilitation of a single tooth with a prosthetic crown supported by implants is one of the most frequently performed procedures, thanks also to the high rate of success documented in the literature [1, 2].

To obtain an optimal functional and aesthetic result, the implant should be positioned considering all three dimensions [3, 4, 5]. It is also fundamental not only that there be an adequate bone architecture but also that the hard tissues are correctly handled [6]. Additionally, obtaining optimal aesthetic results involves correct handling of the soft tissue with adequate surgical and nonsurgical procedures. This becomes even more necessary for the anterior teeth [7], where gross errors impede a satisfactory rehabilitation of the patient. It is crucial to emphasize the importance of manufacturing temporary crowns [8] in order to shape the emergent profiles before proceeding with the definitive restoration. A temporary crown with an adequate contour will shape and stabilize the peri-implant soft tissue.

**Figure 1:**
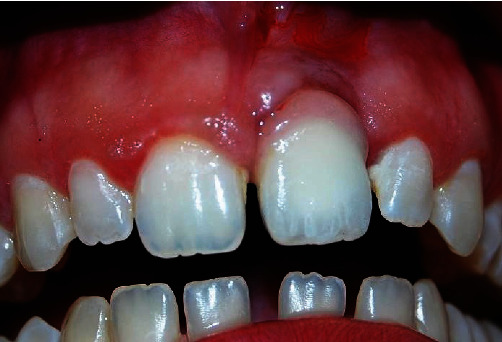
The patient came to us with a prosthetic rehabilitation implant for the 21 that did not satisfy any functional or aesthetic parameter.

The patient who came to our notice had an implant-supported restoration on element 21, which was not satisfying any functional or aesthetic parameter. Furthermore, he presented with an altered passive eruption [9] in the contralateral hemiarch (Figure 1).

Specifically, it was clear that the interdental gingiva was not represented [10], and the emergent profile was altered. A poor management of the soft tissues [11] had led to a nonharmonic integration of the prosthetic crown, and it resulted in an excessive retraction of the tissues. The arches were completely unaligned, resulting in an unacceptable rehabilitation.

Furthermore, during the previous treatment, the altered passive eruption [12] on the contralateral arch was not considered; had it been adequately treated together with the implant-prosthetic rehabilitation, it could have contributed to obtaining a satisfactory aesthetical and functional result, obtaining an adequate realignment of the arch.

## 2. Materials and Methods

**Figure 2:**
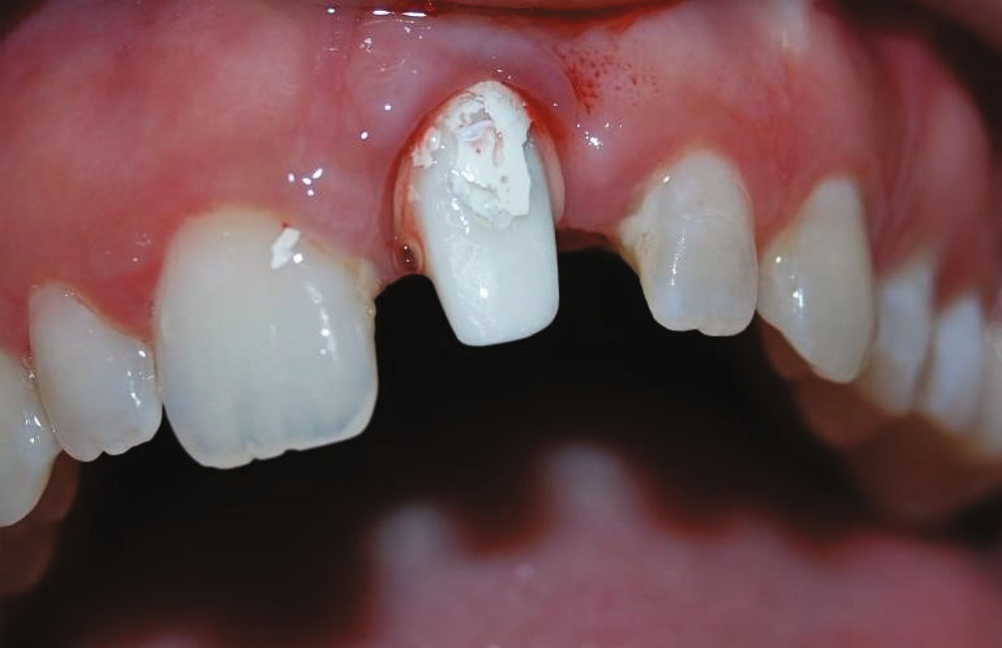
Once the crown was removed, the stump was newly prepared with a diminishing edge in zirconium and a temporary therapeutic element.

The prosthetic crown was removed from the implant, which allowed the study of the peri-implant tissues that were found to be flat and compressed, giving no possibility of reshaping or of adaptation (Figure 2).

Moreover, a horizontal overcontour was emphasized that had contributed to worsening of the aesthetics and the already altered emergent profile, thus not allowing the superficial periodontal tissues to adapt to the prothesis and contributing to the aesthetic deficit [13].

**Figure 3:**
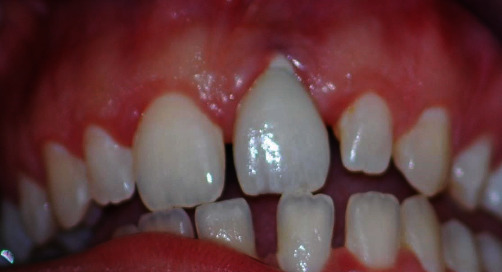
A temporary therapeutic element was inserted.

After removing the crown, a zirconia abutment with a decreasing contour was remade and a provisional restoration was placed leaving 2 mm space between it and the free gingival margin, giving the tissues the possibility of descending around the crown and substituting the missing gingival tissue, consequently allowing integration between the periodontal tissues and the provisional prothesis (Figure 3).

**Figure 4:**
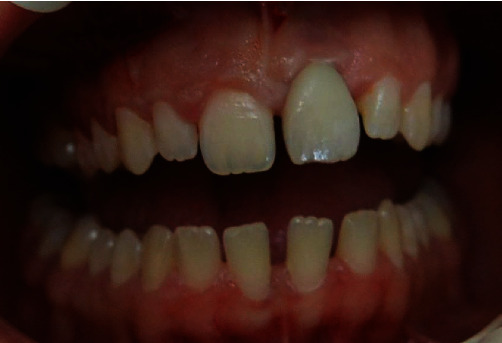
During the day 30 examination it was possible to see that the tissues had descended by at least 1.5 mm and were beginning to define the possibility of a new emergent profile.

After 30 days, the patient was examined, and it was already possible to see that the tissues had descended at least 1.5 mm and that they were defining a new emergent profile, a sign of correct integration between the periodontal area and the prosthetic crown (Figure 4).

The provisional prothesis was then reinserted and the next phase commenced—the redefinition of the arches operating on the altered passive eruption; this allowed functional identical profiles to be obtained, which were also aesthetically pleasant.

A surgical procedure to restore the periodontium was performed, allowing the realignment of the arches; therefore, to resolve the condition of the altered passive eruption, gingivectomy was carried out according to Goldman [14] but with slight modifications. The patient was anesthetized locally using Optocain 20 mg/ml with adrenalin 1 : 100,000, the bleeding points (BPs) at the apex of the sulcus were located, the operational area was then defined by joining the BPs, and the projection of the future parabolas was determined.

**Figure 5:**
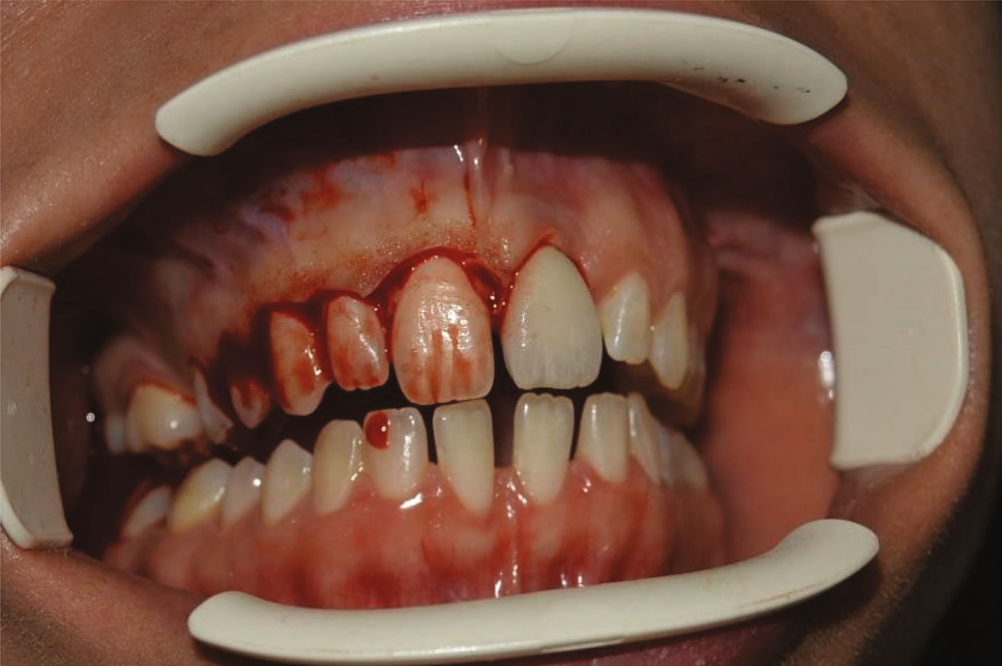
Using a 15c scalpel, a full depth flap was created with a primary nonmarginal incision placed 2 mm from the edge and an intrasulcular fillet incision.

Using a 15c scalpel, a full-thickness flap was performed with a submarginal primary incision at 2 mm from the gingival border, followed by an intrasulcular incision to free the gingival collar, which was then entirely removed with the help of a probe [15, 16] (Figure 5).

At the end of the procedure, the final intrasulcular result could already be envisaged, the new alignment of the emergent profiles, the length of the clinical crown of the dental elements, and the almost perfect adaption of the tissues to the provisional prothesis supported by the implant [17].

Natural, nonabsorbing, monofilament sutures were then applied (Seta 4\0) using separated simple stitches [18].

**Figure 6:**
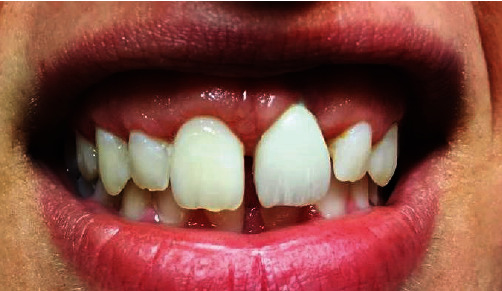
Day 21 examination.

After 10 days, the suture was removed, and after 21 days, the patient was examined to assess the results of the healing process [19] (Figure 6).

**Figure 7:**
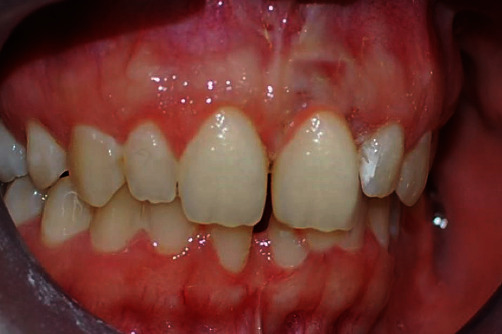
3 months later, the tissues were properly stabilized and the definitive crown was inserted, completing the rehabilitation.

After 3 months [20], the tissues were adequately stabilized and a permanent prosthetic crown was fitted, finishing the rehabilitation process (Figure 7).

## 3. Discussion

Through a periodontal approach, both surgical and prosthetic, the patient was correctly rehabilitated, regaining function and aesthetics, balancing the emergent profiles, making the two hemiarches congruent and the prosthetic crown biomimetic and in harmony with the natural dentition.

The lack of planning or inadequate planning, as in this case, exposes treatments to mistakes, and it is not easy to repair the initial error and also obtain an optimal result. To correctly plan a therapeutic program requires certain considerations.

The clinical cases can regard a single tooth, but they should be always seen as a part of the whole mouth. Thus, in the initial evaluation the occlusion, the relationship between the mandible and the maxilla, and both the periodontal and the functional aspects must be taken into account. This allows clinicians to establish if there is any necessary work to be done on both the hemiarches, as in the described case, and to plan and program the therapeutical plan carefully.

To solve such a clinical case, several easily observable periodontal factors must be considered. In this specific case, considering the severe bone deficit of the patient, a space around the crown should have been left without touching the tissue integration. Furthermore, an altered passive eruption on the contralateral arch was present and the elimination of this factor with a simple gingivectomy, operation that is advisable in this sort of situation, allowed us to restore the emergent profiles, renewing the aesthetics and the congruence of the whole of the patient's mouth.

It is important to emphasize that in an aesthetic rehabilitation trough implant, not only the placement of should the implant be guided prosthetically, but it is indisputably necessary that the clinician should be expert in periodontology so that they can plan and perform, when necessary, all the therapeutical options (surgical and nonsurgical) that will lead to the best possible result [21], especially in complex clinical cases like the one just described.

## 4. Conclusions

This work aimed at showing how to resolve similar cases but also proposing some considerations that are more generally useful:

1. It is a good idea to always plan every therapeutic intervention with awareness, assessing the whole of the patient's mouth and the patient themselves (state of general health, concomitant conditions, etc.) in order to minimize the risks and to have a choice between possible alternatives.
2. Consideration of the periodontal factors when one has to resolve similar situations is fundamental; implantology and prosthetics are disciplines that cannot be separated from periodontology; otherwise, the correct or incorrect tissue adaption of the periodontal tissues is left to chance.

## Data Availability

Data can be provided by requesting the author directly.
